# A Stem Cell Strategy Identifies Glycophorin C as a Major Erythrocyte Receptor for the Rodent Malaria Parasite *Plasmodium berghei*

**DOI:** 10.1371/journal.pone.0158238

**Published:** 2016-06-30

**Authors:** Loukia Yiangou, Ruddy Montandon, Katarzyna Modrzynska, Barry Rosen, Wendy Bushell, Christine Hale, Oliver Billker, Julian C. Rayner, Alena Pance

**Affiliations:** 1 Malaria Programme, Wellcome Trust Sanger Institute, Cambridge, United Kingdom; 2 Mouse Developmental Genetics and ES Cells Mutagenesis, Wellcome Trust Sanger Institute, Cambridge, United Kingdom; 3 Cellular Genetics, Wellcome Trust Sanger Institute, Cambridge, United Kingdom; 4 Microbial Pathogenesis, Wellcome Trust Sanger Institute, Cambridge, United Kingdom; Université Pierre et Marie Curie, FRANCE

## Abstract

The clinical complications of malaria are caused by the parasite expansion in the blood. Invasion of erythrocytes is a complex process that depends on multiple receptor-ligand interactions. Identification of host receptors is paramount for fighting the disease as it could reveal new intervention targets, but the enucleated nature of erythrocytes makes genetic approaches impossible and many receptors remain unknown. Host-parasite interactions evolve rapidly and are therefore likely to be species-specific. As a results, understanding of invasion receptors outside the major human pathogen *Plasmodium falciparum* is very limited. Here we use mouse embryonic stem cells (mESCs) that can be genetically engineered and differentiated into erythrocytes to identify receptors for the rodent malaria parasite *Plasmodium berghei*. Two proteins previously implicated in human malaria infection: glycophorin C (GYPC) and Band-3 (Slc4a1) were deleted in mESCs to generate stable cell lines, which were differentiated towards erythropoiesis. *In vitro* infection assays revealed that while deletion of Band-3 has no effect, absence of GYPC results in a dramatic decrease in invasion, demonstrating the crucial role of this protein for *P*. *berghei* infection. This stem cell approach offers the possibility of targeting genes that may be essential and therefore difficult to disrupt in whole organisms and has the potential to be applied to a variety of parasites in diverse host cell types.

## Introduction

Malaria is a devastating infectious disease caused by *Plasmodium* parasite species that cycle between humans and *Anopheles* mosquitoes. While the parasite’s life cycle is complex, it is the infection of erythrocytes which is responsible for the symptoms and complications of the disease [1, 2]. *Plasmodium* species are obligate intracellular parasites that exist only briefly as an extracellular form, the merozoite, during the blood stages. The process by which merozoites recognise and enter erythrocytes is highly complex and depends on a sequence of steps determined by specific molecular interactions. Initially, attachment to the erythrocyte membrane occurs through ligands distributed across the merozoite surface. A reorientation then places the apical end of the parasite into close contact with the erythrocyte membrane, where a dense junction forms followed by an active entry process [3, 4].

The complexity of the invasion process clearly relies on multiple receptor-ligand interactions between erythrocyte and merozoite, but relatively few such interactions have been identified and characterised at the molecular level. Furthermore, these interactions are likely to be highly species-specific, so what is known about interactions in one *Plasmodium* species cannot be directly transferred to another. Most is known about the parasite that causes the majority of human malaria mortality, *P*. *falciparum*, which uses a number of receptor-ligand interactions to facilitate invasion, some of which appear to be redundant. Its genome encodes multiple members of the erythrocyte binding-like (EBL) and reticulocyte binding-like (RBL) invasion ligand families, which have been identified in every *Plasmodium* species sequenced to date [5]. Receptors have been identified for some of these proteins, such as PfEBA175 which interacts with the predominant erythrocyte surface sialoglycoprotein Glycophorin A [6], PfEBA140 which interacts with Glycophorin C (GYPC), a component of the Gerbich blood group involved in maintaining the shape and membrane properties of erythrocytes [7, 8] and PfRH5 which interacts with basigin, the determinant of the Ok^a^ blood group [9]. By contrast, there is no evidence that many other species including the other most abundant human parasite, *P*. *vivax*, use these proteins for invasion. Instead, this species critically depends on *P*. *vivax* Duffy Binding Protein (PvDBP), which binds to the Duffy Antigen Receptor for Chemokines (DARC) [10, 11]. Though DARC was also shown to be an important mediator of infection by the simian parasite *P*. *knowlesi*[12, 13] as well as the more distantly related rodent parasite *P*. *yoelii* [14], in these species, it is not essential. Therefore, the use of receptors across species cannot be predicted based on phylogenetic distance alone. Other erythrocyte proteins are thought to play a role in invasion, although their function is not always clearly defined. The anion transporter Band-3 (Slc4a1) is a type IV membrane-spanning surface protein that is thought to interact with *P*. *falciparum* MSP1 and MSP9 proteins and therefore may be directly involved in invasion [15, 16] although other roles are also possible. This protein harbours a 9 amino acid deletion in Southeast Asian Ovalocytosis, a condition associated with resistance to cerebral malaria [17–19] although the exact mechanism involved in resistance is not yet clear [20]. Whether Band-3 is important in other *Plasmodium* species is not known, and hard to predict due to the species-specificity of these interactions and for many commonly studied *Plasmodium* parasites, including the widely used rodent model *P*. *berghei*, no receptors have been thus far indetified.

For many commonly studied *Plasmodium* parasites, including the widely used rodent model *P*. *berghei*, no receptors have been thus far indentified. Part of the challenge of identifying receptors is technical. Some biochemical approaches have been attempted, relying on protein binding or co-immunoprecipitation, but the often low affinity of surface protein-protein interactions makes their identification difficult [21]. Other studies used erythrocytes from human genetic variants with specific surface proteins phenotypes, but these are limited by the relatively rare occurrence and scarce availability of such cells [22–24]. Stem cells provide a promising alternative since genetic changes can be introduced during the pluripotent state and the modified cells can then be differentiated into erythrocytes to assess the impact of these changes on *Plasmodium* infection. Pioneering studies testing this approach have been performed using human haematopoietic stem cells (HSCs) in which shRNA knockdown of specific erythrocyte proteins was shown to restrict invasion by *P*. *falciparum*, recapitulating known receptor preferences as well as identifying novel host determinants of the disease [25, 26]. Here we explore the potential of mouse Embryonic Stem Cells (mESCs) as a genetic system to identify host factors involved in the rodent model of malaria using *Plasmodium berghei* infection.

mESCs are diploid pluripotent cells derived from the inner cell mass of the blastocyst at the early stages of embryogenesis. They can be cultured and expanded *in vitro* maintaining their self-renewal and pluripotency indefinitely. Since mESCs are stably kept in the laboratory, permanent genetic manipulation is possible and because they are pluripotent, they have the ability to give rise to all three germ layers of an organism: endoderm, mesoderm and ectoderm [27] and differentiate into various cell types including functional erythrocytes [28, 29]. Here, we combine genetic manipulation of mESCs, erythropoietic differentiation and *Plasmodium* infection assays to investigate host-parasite interactions in the model rodent parasite *P*. *berghei*. We focus on two erythrocyte proteins, Glycophorin C (GYPC) and Band-3 (Slc4a1), aiming to include an abundant membrane protein and structural component of the erythrocyte. These proteins are localised in different complexes on the erythrocyte membrane and though no direct parallels can be drawn, there is evidence for their participation in the invasion process in several *Plasmodium* species. Our results show that while Band-3 does not appear to play a role, GYPC is crucial for *P*. *berghei* invasion.

## Materials and Methods

### Embryonic Stem Cell culture

The JM8.N4 cell line, derived from the mouse strain C57BL/6N [30], was cultured on 0.1% gelatin coated plates (Corning NY, USA) in Knockout DMEM (Gibco, UK) containing 2mM L-Glutamine (Gibco, UK), 0.05mM β-mercaptoethanol (Sigma Dorset, UK), 10% fetal bovine serum (Biosera East Sussex, UK) and 1000U/ml LIF (ESGRO, Millipore Watford, UK), at 37^0^ and 5% CO_2,_ changing medium daily. Cells are detached with 0.1% trypsin (Gibco, UK), containing 1% chicken serum (Gibco, UK), 0.6 mM EDTA (Sigma Dorset, UK) and 0.1% D-glucose (Sigma Dorset, UK) and passaged every two to three days. The E14 mESC line (129P2 background) was cultured in the same way but using GMEM with the addition of pyruvate and keeping the cells in 7% CO_2._

### Embryoid Body formation

Cells were re-suspended (2x10^6^cells/ml) and cultured in Hyclone IMDM (Thermo Scientific, UK) with 15% Fetal Bovine Serum, 10% PfHMII (Gibco, UK), 2mM L-Glutamine solution (Gibco, UK), 300μg/ml Holo-Transferrin (Sigma Dorset, UK), 50μg/ml Ascorbic Acid (Sigma Dorset, UK), 0.4mM MTG (Sigma Dorset, UK) and 1ng/ml mouse IL-3 (Gibco, UK) [28], in 100mmx20mm bacterial plates (Sterilin Newport, UK). EBs were cultured for 6 days, adding fresh medium half way through.

### Erythroid Differentiation

EBs were dissociated by trypsinisation and the resulting single cell suspension taken through a 3 step differentiation protocol [29] as follows:

#### Expansion medium I

StemPro®34 SFM (Gibco, UK) with 1μM Dexamethasone (Life Sciences, UK), 1.5U/ml EPO (Sigma Dorset, UK), 100ng/ml mouse SCF (BioLegend London, UK), 40ng/ml mouse IGF1 (Shenandoah Biotechnology Inc Warwick, UK) and 10ng/ml mouse IL-3 (Gibco, UK). Cells were cultured at a density of 10^6^cells/ml for 4 to 5 days.

#### Expansion medium II

StemPro®34 SFM (Gibco) with 1μM Dexamethasone (Life Sciences, UK), 1.5U/ml EPO (Sigma Dorset, UK), 100ng/ml mouse SCF (BioLegend London, UK) and 40ng/ml mouse IGF1 (Shenandoah Biotechnology Inc Warwick, UK). Cells were cultured for 4 days or until they stopped proliferating, centrifuged at 200 xg for 3 minutes at room temperature and washed x3 with sterile PBS before changing into the differentiation medium.

#### Differentiation medium

StemPro®34 SFM (Gibco, UK), 10U/ml erythropoietin (Sigma Dorset, UK), 40ng/ml mouse IGF1 (Shenandoah Biotechnology Inc Warwick, UK), 0.5µg/ml human Holo-Transferrin (Sigma Dorset, UK) and 10μg/ml human Insulin. Cells were counted on a regular basis using a hemocytometer and diluted 1:10 in Hyclone Trypan Blue Solution (Thermo Scientific, UK).

### RNA extraction and RT-PCR analysis

Total RNA was extracted using the Bioline (London, UK) isolate RNA kit following the manufacturer’s instructions and quantified on a NanoPhotometer®. RNA (1–3μg) was reverse transcribed with a MuLV reverse transcriptase (Applied Biosystems, UK) and random primers (Bioline, UK). One μl of cDNA was specifically and quantitatively amplified using the SybrGreen system (Applied Biosystems UK) following the established cycling parameters and using actin as a control for normalisation. Primers used (5’-3’):

Actin           F-CACCCTGTGCTGCTCACCGAG           R-GGATGGCTACGTACATGGCTGGG

Oct4           F-GACGCGAAGATCTGCAACTG              R-GCTGCGAGTACACATTGAGG

Nanog         F-GCTACTGAGATGCTCTGCAC              R-GTCAGCCTCAGGACTTGAGAGC

GATA1       F-GCAGCATCAGCACTGGCCTAC            R-GGTAGAGTGCCGTCTTGCC

Brachyury    F-GAGAGCGAGCTGTGGCTGCG           R-GAGTACATGGCATTGGGGTCCAGG

Tal1             F-GCGCTGCTCTATAGCCTTAGCCAG    R-CCGGTTGTTGTTGGTGAACATGG

CD34          F-GTAGCTCTCTGCCTGATGAG               R-CCTGATAGATCAAGTAGTGGC

GYPA         F-CATTCACCATCATTGTCAGGATCAG   R-GACACTTCAGTAGGGGCCGTGTG

Hbα             F-GGAAGATTGGTGGCCATGGTG           R-GACCTGGGCAGAGCCGTGGC

Hbβ             F-GGTGCACCTGACTGATGCTGAG        R-CCGCTGGGTCCAAGGGTAGAC

Hbγ             F-GACAACCTCAAGTCTGCCTTGGC     R-CAATCACCAGCACATTACCCAAGAG

GYPC         F-GATCACTGCTGTGGCCTTGGTC          R-CTCTGTGCCTTTGGCCTCAT

### Flow Cytometry (FACS) analysis

Pluripotency analysis was performed with the BD Stemflow™ Human and Mouse Pluripotent Stem Cell Analysis Kit (BD Biosciences Oxford, UK). Specific surface markers were assessed with fluorochrome-labelled specific antibodies after blocking Fc non-specific interactions by incubation with an anti-CD16/32 antibody for 10–15 minutes. Parameters for analysis were set with unlabelled cells and gating with isotype controls for the appropriate fluorochromes. Compensation for spectral overlap was done when required using BD™ CompBead Plus positive and negative beads. Analysis was run on a LSFORTESSA BD cytometer using Flowjo (7.6.5). Antibodies: CD71-FITC; CD117-APC; CD41-FITC; CD33-PE (BD pharmingen); CD61-PE (Caltag); CD45-Pacblue (biolegend); CD34-FITC (Cambridge Bioscience); CD14-APC (ebioscience)

### Haemoglobin staining

Cells smeared on glass slides were fixed with methanol and incubated in the stain solution (40% ethanol, 0.01M sodium acetate, 0.65% hydrogen peroxide and 0.6 mg/ml o-dianisidine SIGMA) for 15 minutes at room temperature. The slides were washed with water and imaged on a light microscope (Leica).

### *P*. *berghei* schizont culture and purification

All animal procedures were conducted under UK Home Office project licence and approved by the ethics committee of Wellcome Trust Sanger Institute. The experimental protocols performed during this work were classified as "mild" according to the severity categories guidance (Home Office,UK). All animals were monitored daily and showed no symptoms of the malaria infection or any other health issues. The biological material was harvested on the final day under terminal anaesthesia and animals were euthanised immediately afterwards.

The *P*. *berghei* ANKA (line 1804cl1) parasites that express mCherry under the Hsp70 promoter, received from Dr. Chris Janse (Leiden malaria research group, the Netherlands) was used for these studies. C57BL/6N mice were pre-treated with 25mg/ml phenylhydrazine to enrich for reticulocytes and, two days later, injected with 10^7^ mCherry-*P*. *berghei* parasites. Blood was collected and used for schizont culture as described previously [31]. Parasites purified on a Nycodenz gradient were re-suspended in differentiation medium, assessed by microscopy and mechanically ruptured to release the merozoites.

### Invasion Assay

Prior to the invasion assay, ‘target’ cells can be stained with carboxy-DFFDA,SE dye (CellTrace^TM^ Invitrogen, UK). Cells were centrifuged at 1250xg for 3 minutes, washed once and incubated in differentiation medium containing 5μM DFFDA-SE for 2 hours at 37°C. The cells were washed twice and incubated for a further 30 minutes at 37°C. After a short spin as above the cells were resuspended in fresh medium, ready for the invasion assay.

Differentiated cells were centrifuged at 1250 xg for 5 minutes. 5x10^5^ cells were resuspended in 50μl of medium and dispensed in one well of a rounded bottom 96 well sterile plate (Corning NY, USA). To this culture 5x10^5^ ruptured schizonts were added to a final volume of 100μl. The plate was gassed with malaria gas (1% O_2_, 3% CO_2_, 96% N_2_; BOC Guildford UK) in a closed chamber and incubated at 37°C over 24 hours. Mouse uninfected blood was used as a control for each experiment.

### Genomic manipulation of mouse ESCs

Conditional heterozygous knockout mESC lines were obtained from the European Conditional Mouse Mutagenesis Program (EUCOMM) resource [32]. Targeting was confirmed with specific PCR primers and by the Taqman^®^ Copy number assay or loss of allele (LOA) assay [33]. Additionally, euploidy of the most frequently aberrant chromosomes 1, 8, 11 and Y was confirmed.

The second alleles were targeted with final vectors assembled from intermediate constructs from the EUCOMM resource: a custom L1L2 containing β-actin promoter-blasticidin followed by a 3’ loxP site (gift from Peri Tate, WITSI) and L3L4 backbone. Gateway reactions were assembled and transformed into Cre expressing *E*. *coli* as described previously [33]. Cre-recombinase was induced with a heat-shock the next morning at 42°C for 30 minutes and the bacteria were plated on YEG-Cl agar with 0.4% glucose and 50μg/ml spectinomycin. Colonies were picked and the final vector from which the *loxP*-flanked critical region is deleted was extracted and confirmed by restriction digests. The final vectors were prepared and electroporated into mESCs following the above protocol. Colonies were selected with 100μg/ml G418 as well as 1μg/ml blasticidin (Fisher BioReagents, UK) for selection of homozygous cells. Selected colonies were expanded and genotyped.

### Western Blot

Western blots were run as described previously [34] and Glycophorin C expression was assessed with and anti-GYPC antibody (PAB711Mu01, Cloud-Clone Corp, USA) and an HRP-conjugated goat anti rabbit secondary antibody (SIGMA).

### Confocal microscopy

After invasion, the samples were fixed in 4% paraformaldehyde (Fisher Scientific, Loughborough UK) for 20 minutes, washed with PBS and permeabilised with 0.1% TX100 in PBS for 10 min. Blocking was done with 3% BSA in PBS for 30 min followed by an anti-MSP1 antibody, generously donated by Dr. A. Holder [35] for 2 hours. After washing x3 in PBS-3% BSA the cells were incubated with a secondary Alexa Fluor 488-conjugated goat anti-mouse IgG (Invitrogen, USA) for 1 hour and washed x3 again. The cells were smeared on glass slides, mounted using prolong gold DAPI medium (Invitrogen USA) and left to set over night. Slides were analysed on a confocal microscope (Zeiss LSM 510 meta).

### Statistical analysis

Values are presented as means and standard deviation. Any reported significance was calculated using a two-tailed unpaired Student’s t-test analysis.

## Results

### mESCs differentiate into erythroid cells *in vitro*

**Fig 1:**
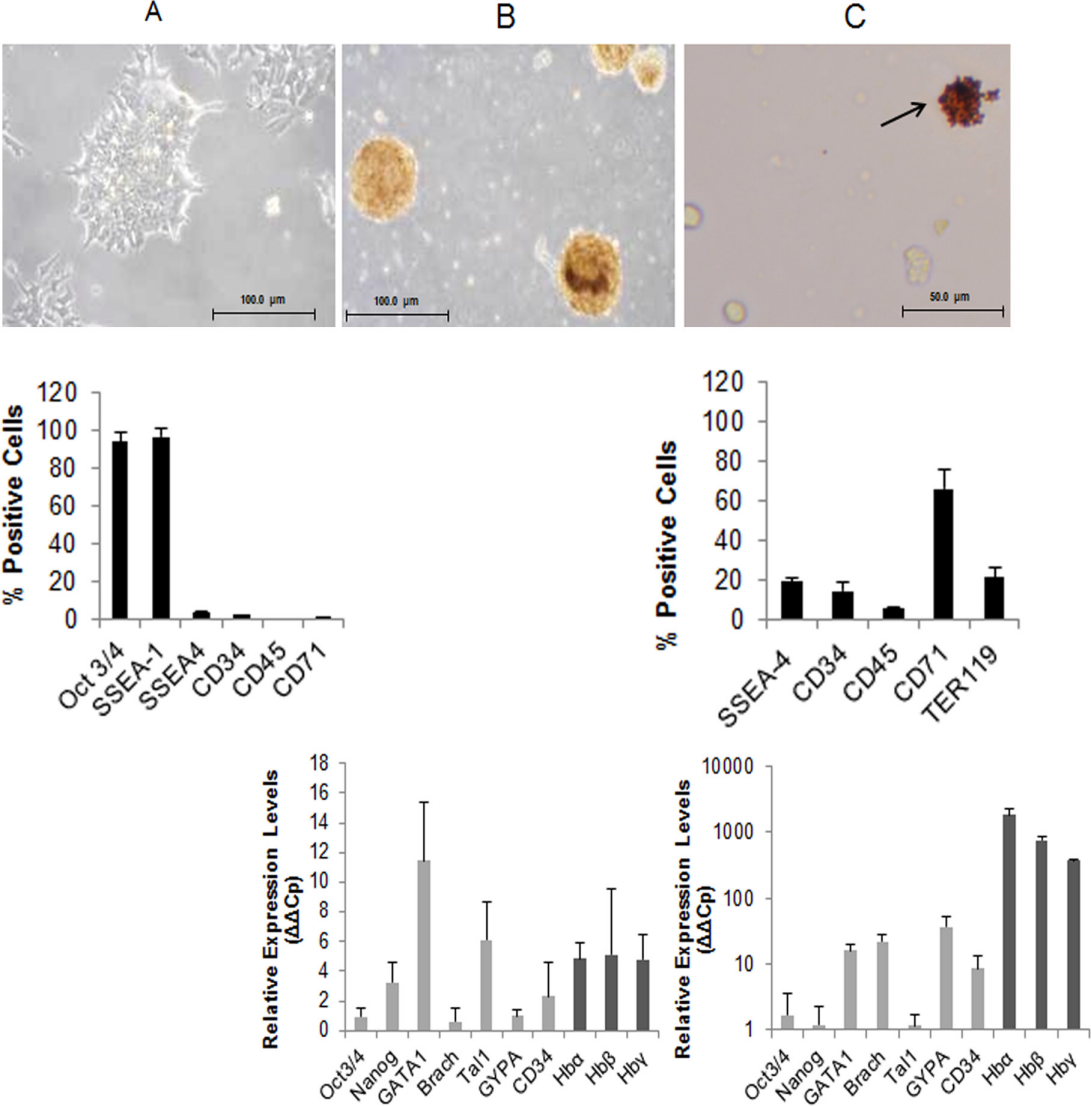
Differentiation of JM8 mESCs towards erythropoiesis. A) **top panel**: pluripotent JM8 cell growth under light microscopy; **middle panel**: analysis of the indicated cell surface markers by flow cytometry. B) **top panel**: morphology of embryoid bodies under light microscopy; **bottom panel**: analysis of haematopoietic markers by qRT-PCR. C) **top panel**: differentiated erythroid cells under light microscopy, black arrow indicates haemoglobinised colony of cells; **middle panel**: analysis of cell surface markers by flow cytometry; **bottom panel**: haematopoietic proteins by qRT-PCR. (Data are presented as mean +/-SD).

The mouse embryonic stem cell (mESCs) line JM8.N4, derived from the mouse strain C57BL/6N was differentiated into erythroid cells *in vitro* using a protocol based on previously described work [28, 29]. Feeder-free JM8 ESCs normally grow in colonies (Fig 1A top panel) expressing the pluripotency markers Oct3/4 and SSEA-1 while very low levels of the general differentiation marker SSEA-4 are detected. Specific haematopoietic markers such as CD34, CD45 and CD71 are also very low at this stage as measured by flow cytometry (Fig 1A middle panel).

The first step of the differentiation protocol is the formation of embryoid bodies (EBs) consisting of tight three-dimensional aggregates of cells (Fig 1B top panel). At this stage, expression of pluripotency markers decreases and concomitantly some of the crucial mesodermal and haematopoietic regulatory transcription factors such as GATA-1 and Tal-1 become more prominent, as measured by qRT-PCR (Fig 1B bottom panel).

**Fig 2:**
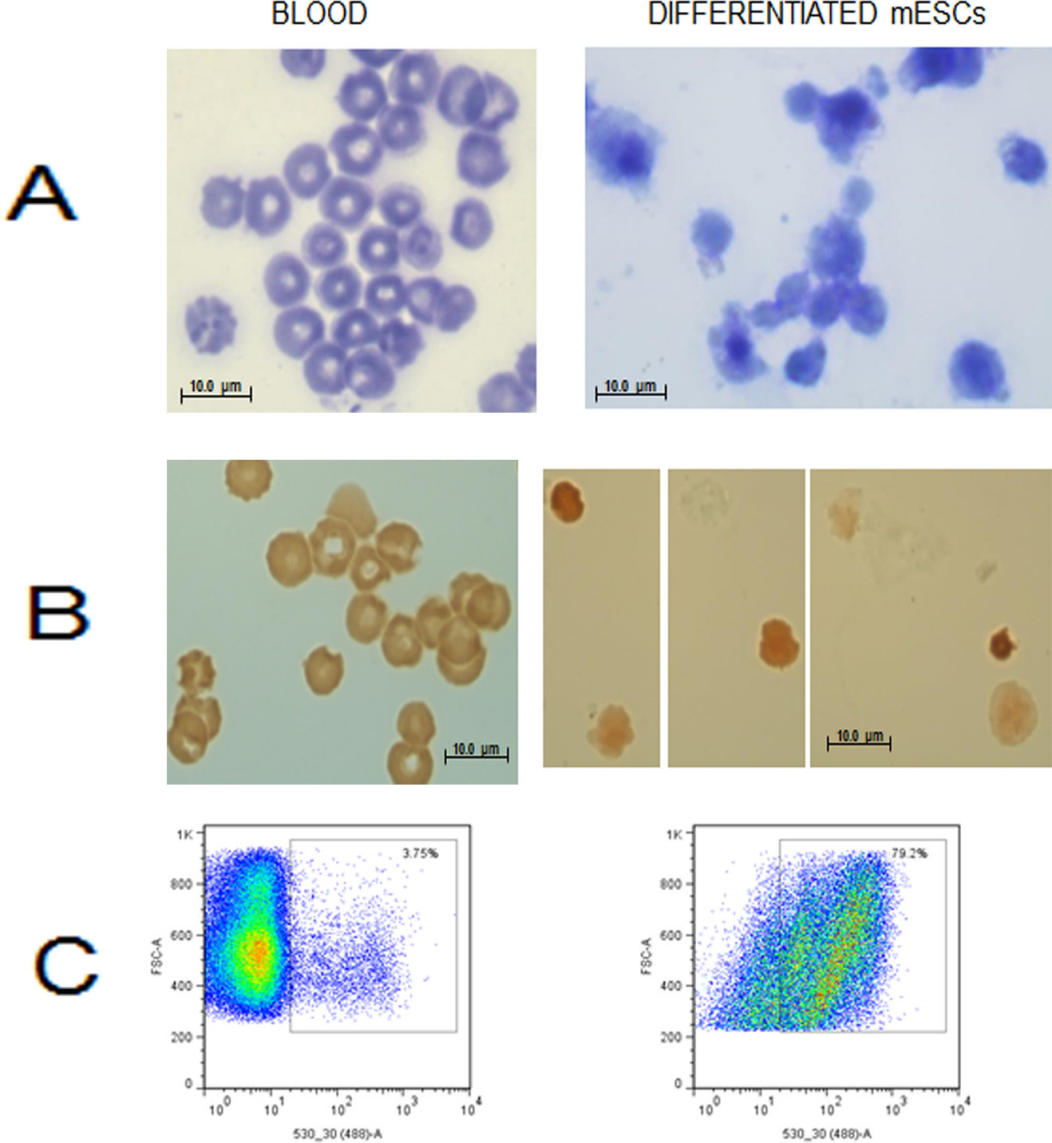
Analysis of differentiated JM8 erythroid cells. A) Rapid Romanowski stain of mouse blood and differentiated cells. B) Staining Haemoglobin in mouse blood and differentiated cells with o-dianisidine. C) Flow cytometry detection of nuclei in blood and differentiated cells labelled with Hoechst 33342.

Subsequently, the EBs are disaggregated into a single cell suspension which is taken through a three step differentiation protocol to generate erythroblasts and finally erythroid cells (Fig 1C top panel). Flow cytometry analysis of the cell population for haematopoietic markers shows no significant differentiation towards the myeloid (CD14, CD33) nor megakaryocytic (CD41, CD61) cell types and a small persistence of haematopoietic progenitor cells of approximately 10% witnessed by CD117 labelling (not shown). On the other hand, erythroid markers CD71 and TER119 show increased expression (Fig 1C middle panel). Of particular importance is CD71 that reveals erythroid cells in earlier stages of differentiation which are known to be preferentially infected by *P*. *berghei* [36]. Efficient definitive erythroid differentiation is demonstrated by a high increase in the transcription levels of adult globin genes (haemoglobin-α and β) as shown by qRT-PCR (Fig 1C bottom panel). Morphologically, rapid Romanowski stain reveals a heterogeneous population in the *in vitro* differentiated cells (Fig 2A) in which haemoglobinisation is confirmed by 0-dianisidine staining (Fig 2B). Flow cytometry analysis shows prevalence of nucleated cells (Fig 2C) indicating that the differentiated cells are mainly at a stage of erythroid progenitors.

### *In vitro* differentiated mESCs support *P*. *berghei* infection

In order to assess infection of differentiated cells *in vitro*, we developed an invasion assay adapting previously described protocols [37, 38]. Briefly, *P*. *berghei* schizonts of the ANKA 1804cl1 line that express mCherry under the Hsp70 promoter, are purified, mechanically disrupted and mixed with differentiated cells or blood. The cultures are then incubated for 24 hours under gas and temperature conditions suitable for parasite development. This time period covers the whole intraerythrocytic cycle of *P*. *berghei* during which samples are taken at 6 and 24 hours to measure initial invasion and subsequent development.

**Fig 3:**
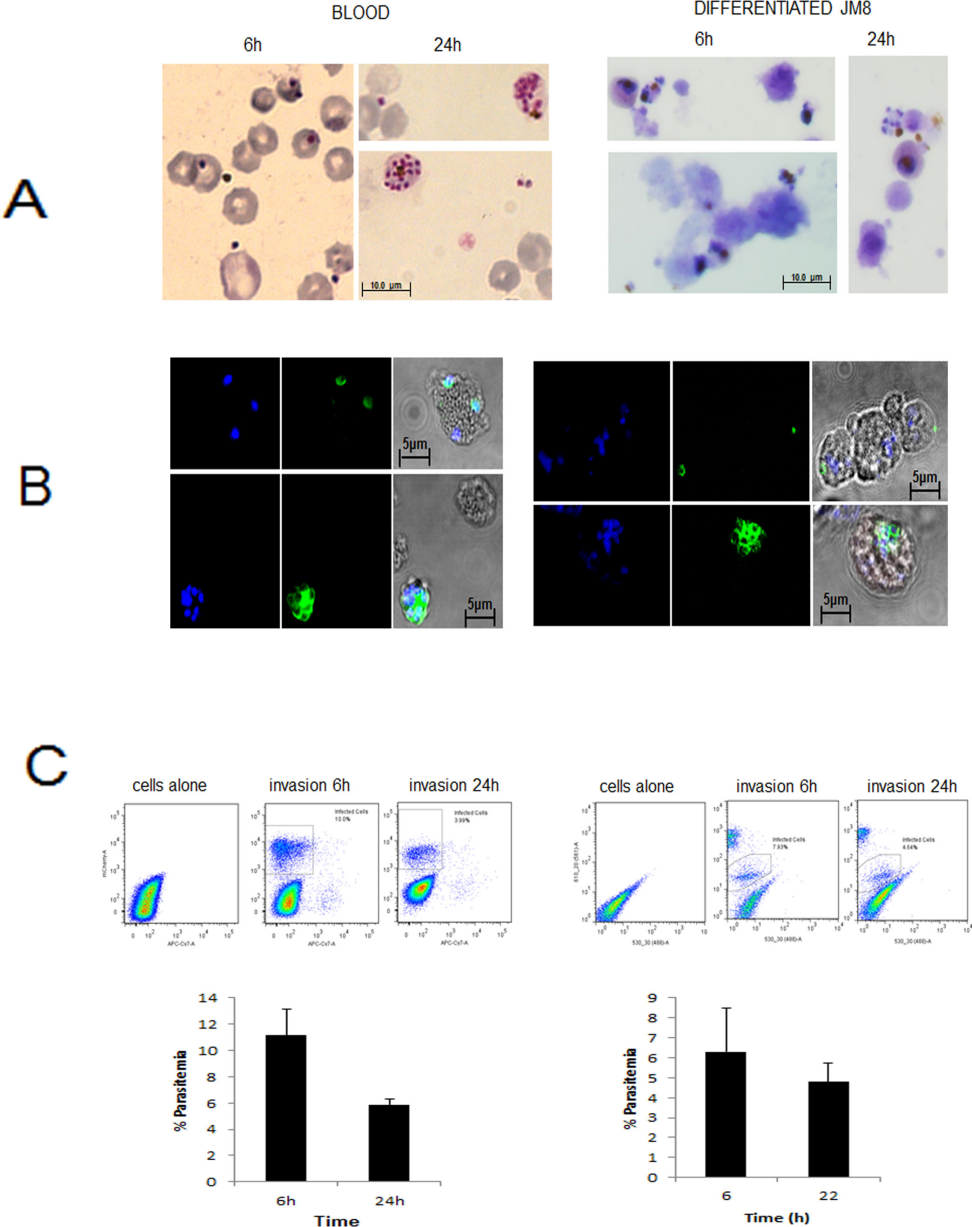
Invasion of *in vitro* differentiated erythroid cells by *P*. *berghei*. A) Rapid Romanowski staining of infected red blood cells and mESC-derived erythroid cells 6 h and 24 h post-infection. B) Confocal microscopy of mouse erythrocytes and *in vitro* generated erythroid cells infected with *P*. *berghei*. Parasites are labelled with an anti-MSP1 antibody followed by a AF488-conjugated secondary antibody and nuclei are stained with DAPI. Invasion is followed at 6 and 24 hours. C) Flow cytometry quantification of infected cells by detecting mCherry-expressing parasites. The plots show the populations distribution for an example experiment and the bar charts are the result of at least three separate experiments. Data are presented at mean +/-SD.

At the first time point of 6 hours, Romanowski staining shows ring stage parasites in the differentiated cells, morphologically similar to those observed in mouse blood (Fig 3A). The 24 hour time point reveals the development of schizonts that are also similar between the differentiated cells and mouse blood. The presence of parasites inside differentiated mESCs was confirmed by MSP-1 staining with specific antibodies and compared to parasites in *in vitro* infected primary mouse erythrocytes (Fig 3B). A proteolytic fragment of this merozoite surface protein (MSP1-19) is carried into the infected erythrocyte and recognised by the monoclonal antibody [35]. At 6 hours post-infection, the expected small ring pattern of MSP1 staining around the parasite is visible, while at 24 hours parasites with the characteristic morphology of schizonts, can be detected.

The use of mCherry-expressing parasites allows invasion events to be quantified using flow cytometry (Fig 3C). Debris and doublets were excluded from the analysis using FSC-A/SSC-A followed by FSC-A/FSC-W and SSC-A/ SSC-W to establish gating of the populations (an example of the gating strategy is shown in S1 Fig, all samples were analysed in the same way). The infection levels detected in the differentiated cells are similar to those observed in mouse blood. In both types of host cells infection levels decrease at the late time point of 24 hours presumably due to the degeneration of some of the formed schizonts together with the known inability of *P*. *berghei* to re-invade *in vitro*.

### Genome editing of mouse ESCs can target genes implicated in malaria infection

To test whether this stem cell system could be used to identify genes involved in *P*. *berghei* invasion, two genes were targeted in mESCs which encode two major components of the erythrocyte membrane: the Band-3 anion transporter (Slc4a1) and Glycophorin C (GYPC).

JM8.N4 cell lines in which one allele of GYPC had been targeted were obtained from the European Conditional Mouse Mutagenesis Program (EUCOMM) resource [32]. The ‘knockout first allele’ in the EUCOMM resource has a gene trap reporter/selection cassette introduced in an intron immediately before a critical exon (exon 2), that forces premature transcription termination of the targeted gene resulting in a null allele (S2A Fig). The EUCOMM cell line was re-cloned and targeting of the first allele was confirmed by the Taqman^®^ Copy number assay (loss of allele assay, LOA) and PCR (S2A Fig). Making use of the modular design of the intermediate targeting vectors from the EUCOMM resource, we generated a construct for homologous recombination into the second allele of GYPC from which the critical exon 2 has been deleted (S2B Fig). The blasticidin resistance gene (BlastR) was introduced as an additional selection marker to allow recovery of double targeted clones, which were verified by LOA (S2B Fig) [39]. Ablation of transcription as well as protein synthesis was confirmed by qRT-PCR and Western Blot (S3 Fig).

This strategy was unsuccessful for the targeting of Slc4a1 in the JM8 ESC line due to genomic instability resulting in a high trisomy frequency of chromosome 11 in which Slc4a1 is located. We obtained from Skarnes and collaborators E14 mESC lines (129P2 background) in which Slc4a1 has been deleted. These lines were constructed using CRISPR/Cas9 technology to induce damage in both alleles of the gene caused by NHEJ. Correct targeting of the resulting clones was verified by Sanger sequencing that showed a 1bp insertion in clone D06 and a 1bp deletion in clone F06, causing a frameshift (S4 Fig). This intervention obliterated transcript and protein synthesis as confirmed by qRT-PCR and flow cytometry (S5 Fig).

### Genetically modified ESCs can be differentiated into erythrocytes

Pluripotency of the modified mESC lines was confirmed by FACS and qRT-PCR (not shown). The lines were differentiated following the protocol described above in parallel with the corresponding wild type mESCs as controls. Inactivation of the target genes in each line was confirmed in the differentiated cells by qRT-PCR (S2 Fig). As expected, no transcript was detected in pluripotent cells, while once differentiated into erythrocytes, high expression was observed in the wild type lines. Only background mRNA levels were detectable in cells differentiated from the GYPC-/- and Slc4a1-/- lines, confirming the successful deletion of the target genes, and by inference absence of the translated protein.

**Fig 4:**
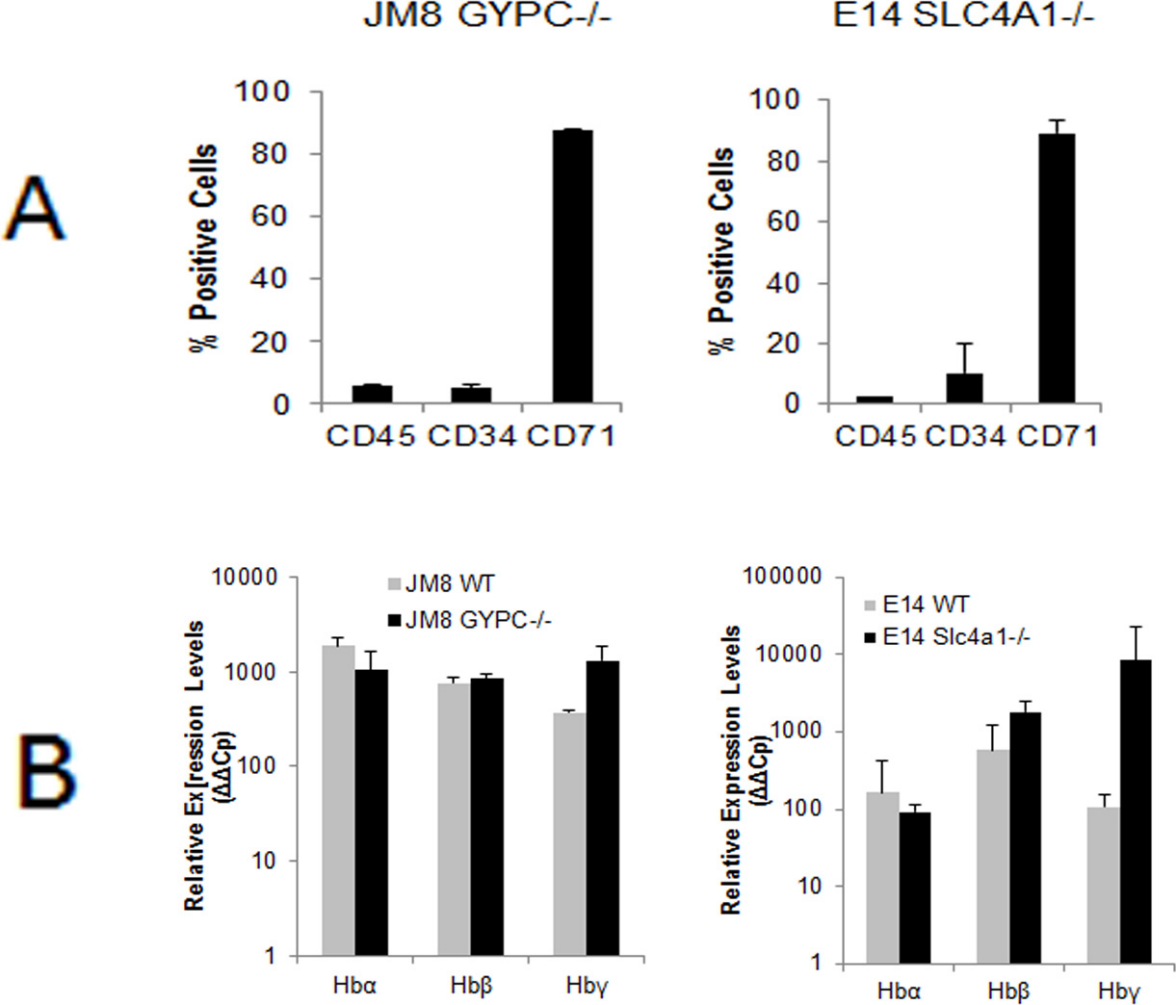
Eyrthropoietic differentiation of genetically modified cell lines. A) Flow cytometric quantification of haematopoietic markers in the differentiated GM lines. B) qRT-PCR quantification of haematopoietic protein transcripts in differentiated GM lines. Data are presented as mean +/-SD. See also S1 Fig.

Differentiation of the KO lines generates a heterogeneous population of cells similar to that observed with differentiated wild type cells. Expression of haematopoietic markers as assessed by FACS shows increased CD34 as well as high levels of CD71 on the membrane of both differentiated KO lines (Fig 4A). A high induction of the adult haemoglobins α, β and γ, similar to the levels observed in the wild type cell lines is detected confirming successful differentiation towards erythropoiesis (Fig 4B).

### Gene disruption generates different phenotypes of infection

**Fig 5:**
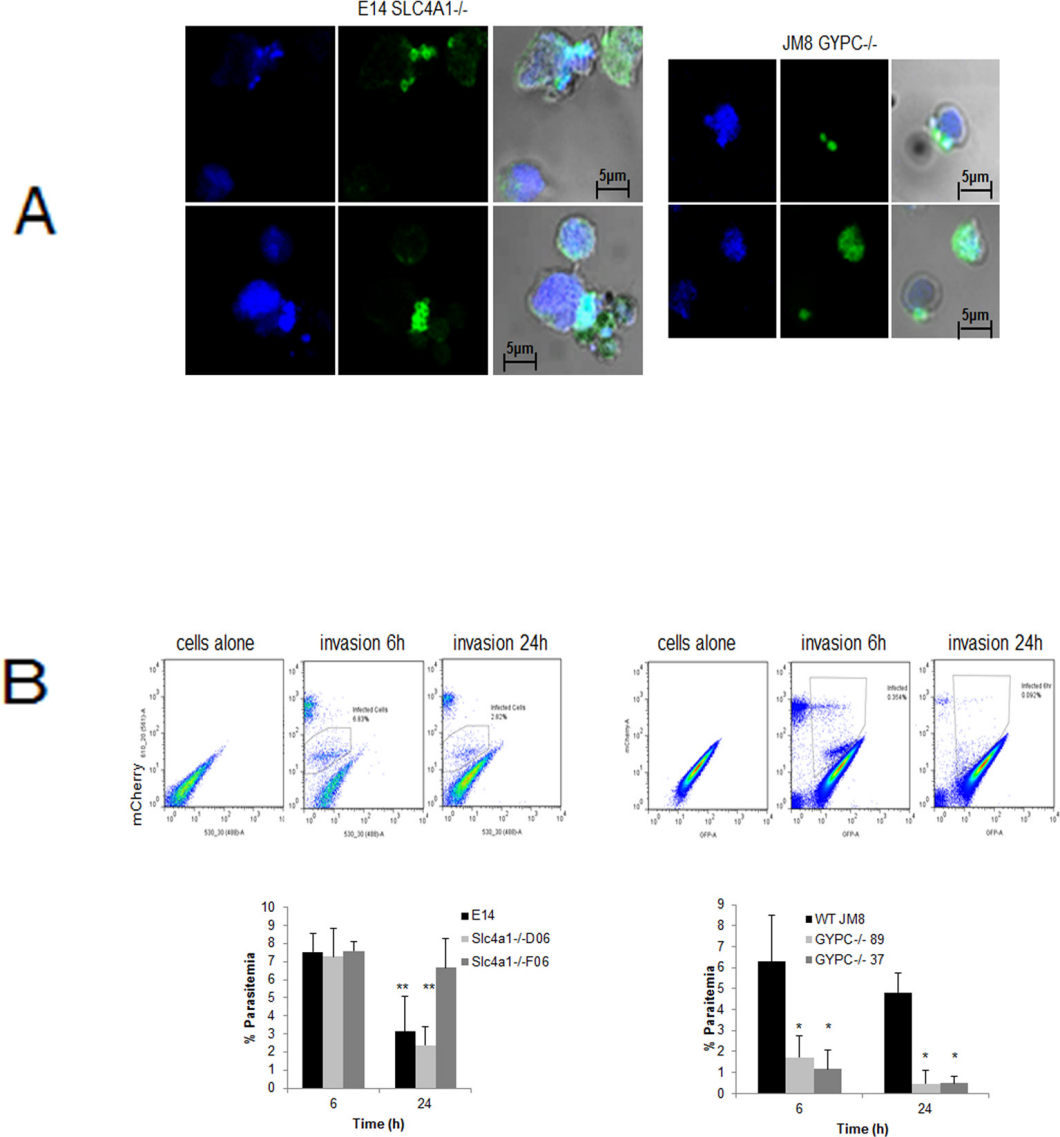
Infection of differentiated genetically modified cell lines with *P*. *berghei*. A) Infection of Slc4a1-/- and GYPC-/- differentiated cells. Labelling with MSP-1 and confocal microscopy showing parasites in the cells. B) Flow cytometric quantification of m-cherry-parasite positive cells at 6 and 24 hours. The plots show the populations distribution for an example experiment and the bar charts are the result of at least three separate experiments (* P<0.01 and ** P>0.1 comparing the clones at each time point with WT). Data are presented as mean +/-SD. See also S6 Fig.

Differentiated wild type E14 and JM8 ESCs as well as the Slc4a1-/- and GYPC-/- lines derived from them, were infected with *P*. *berghei in vitro*. Confocal immunofluorescence microscopy confirmed MSP1 labelling characteristic of rings at 6 hours post-infection and the presence of some schizonts at the end of the 24 h cycle in the KO cell lines for both genes (Fig 5A), though the frequency and size of the parasites is smaller in the GYPC-/- cell line (Fig 5A right panel).

Two independently generated clones for each inactivated gene were tested for their ability to support *P*. *berghei* infection. Quantification by flow cytometry shows that inactivation of Slc4a1 does not affect the capacity of *P*. *berghei* to infect these cells (Fig 5B left panel). In order to verify that the edited differentiated cells are indeed invaded by the parasite, they were labelled with the cell tracker DFFDA-SE before the invasion assay (S6 Fig). Counting of double-labelled events, representing DFFDA-cells infected with mCherry-parasites, confirms that *P*. *berghei* is capable of invading *in vitro* differentiated Band-3 KO mESCs. Though survival of the parasites in the Slc4a1-/- cells seems higher at 24 hours, this effect is not significant (P>0.1). In marked contrast, the ability of GYPC KO cells to support *P*. *berghei* infection is dramatically reduced by 66% at 6 hours and by 86% at 24 hours (P<0.01) (Fig 5B right panel), indicating that this is an important receptor for the rodent parasite, just as it is for *P*. *falciparum* [7, 8].

## Discussion

In this work we use a stem cell-based strategy to investigate host factors of a rodent malaria model. With this technology we are able to generate genetically edited erythroid cells that support infection with *P*. *berghei in vitro*. These are largely immature nucleated cells that may differ in some aspects from erythrocytes, just as most *in vitro Plasmodium* culture systems do not reflect the true diversity and heterogeneity of the host cells encountered by parasites *in vivo*. However, just as the latter, the stem cell-based strategy describe here offers great potential for the understanding of the host proteins implicated in malaria infection. We show that inactivation of Band-3 in mouse erythrocytes does not affect invasion by *P*. *berghei*, in strong contrast to infection by *P*. *falciparum* which is inhibited by deficiency of this protein (reviewed in [40]). This emphasises the specificity of host-parasite interactions and the difficulties of inferring function from one *Plasmodium* species to another at such a rapidly evolving interface. The ability to examine the function of Band-3 in a knock out cell line highlights the potential of this strategy, which allows manipulation of essential genes that are lethal in whole organisms or inaccessible in terminally differentiated cell types devoid nuclei. Homozygous mutations affecting the function of Band-3 are not known to occur naturally and though a KO mouse for Band-3 has been generated, 85 to 90% of the mice die neonatally. The surviving animals suffer extreme anaemia, low body weight and splenomegaly [41, 42], which makes their use for malaria infection studies very difficult. In humans, mutations leading to a decrease of Band-3 expression are found exclusively in heterozygosis [43], and some (as the Southeast Asian Ovalocytosis) have been linked with resistance to malaria infection [17–19]. Only one case of a homozygous mutation (Coimbra) leading to a complete absence of Band-3 has been described in a human patient [44], with reported serious health conditions and dependency on systematic transfusion. Furthermore, Band-3 forms a membrane complex with other proteins such as glycophorin A (GYPA), Rh and CD47 on the erythrocyte plasma membrane. In studies of erythrocytes from the Coimbra mutation patient it was noticed that the loss of Band-3 is accompanied by a loss of membrane expression of the other members of this complex including GYPA [44, 45]. The same dependency of Glycophorin A on Band-3 membrane localisation was observed in mouse erythrocytes [46] and implies that *P*. *berghei* does not depend on GYPA to invade mouse erythrocytes, contrary to *P*. *falciparum* which is well known to use GYPA as one of its main invasion receptors [6]. This is consistent with previous data showing that the murine *Plasmodium* parasites *P*. *berghei* and *P*. *chabaudi* do not require GYPA to invade mouse reticulocytes [47].

In human erythrocytes GYPC is part of a protein complex distinct from the Band-3-GYPA complex, so GYPC membrane localisation is not affected by deficiency of Band-3 [45]. Our data shows that knock out of GYPC dramatically decreases invasion, indicating that, in contrast to Band-3 and GYPA, this protein is crucial for *P*. *berghei* infection. GYPC is also implicated in invasion by *P*. *falciparum*, but it appears to play a less important role. *P*. *falciparum* uses several pathways to invade human erythrocytes [48, 49] and blockage of the GYPC pathway is most effective when others are inhibited at the same time (reviewed in [50]). The dramatic decrease in *P*. *berghei* infection of GYPC-/- differentiated cells suggests a much heavier dependency on GYPC for invasion in this species and that alternative invasion pathways may be either missing or less important. *P*. *falciparum* recognises human GYPC through PfEBA140, a member of the EBL ligand super-family. The *P*. *berghei* genome contains one EBL family protein with homology to PfEBA140 (PBANKA_1332700) which is a clear candidate for interaction with GYPC. However, there are several members of the RBL ligand super-family in the *P*. *berghei* genome that could alternatively be binding partners.

## Conclusion

Taken together, our results highlight species-specific differences in invasion pathways and identify a major receptor for *P*. *berghei*. Our work shows that stem cells are a useful model for the analysis of the host components of infectious diseases and strengthens the field together with other studies that have used haematopoietic stem cells to knock down erythrocyte surface proteins to identify host factors required for *P*. *falciparum* invasion [26]. The system presented here has the advantage of generating stable cell lines with permanent genetic changes in a stable background that can be repeatedly used as a continuous source for study. On the other hand, as the erythroid cells are differentiated *in vitro*, they are nucleated and physiologically distinct from primary erythrocytes, which could influence their interation with the parasite and have an impact on the infection outcome. The development of new mutagenesis technologies that increase the efficiency and specificity of targeting, especially the CRISPR/Cas9 system, further increases the value of this strategy. Targeting of difficult and essential genes becomes possible achieving a clean and complete inactivation resulting in a true null phenotype difficult to obtain with other technologies. Of particular interest for future development are inducible systems that allow ‘turning off’ the desired genes at specific times, for example once the cells are differentiated. We demonstrate that various ESC lines from different backgrounds can be differentiated towards erythropoiesis, which also opens the possibility of exploiting existing resources of knock out mice as a supply of stem cell lines to be analysed in this way. Furthermore, since stem cells are pluripotent, provided good differentiation protocols can be designed, this strategy could be exploited to explore other stages of the pathogen’s life cycle that are normally inaccessible *in vitro*, such as the liver stages in the case of *Plasmodium* parasites [51]. Finally, as human pluripotent cells also have the capacity to differentiate to the haematopoietic lineage [52], extending our approach to a direct functional interrogation of human parasites such as *P*. *falciparum* is a clear and exciting possibility.

## Supporting Information

S1 Fig Flow cytometry gating strategy. A) Debris was eliminated using SSC-A/FSC-A scatter plots. Doublets were excluded using B) FSC-W/FSC-A and C) SSC-A/SSC-W plots.
(TIF)

S2 Fig Genotyping of the Glycophorin C deletion clones. A) Diagram of the vector used to target the first allele. The colonies recovered were tested for vector integration by PCR, using the indicated primers. The copy number of the gene of interest was determined by Loss Of Allele assay (LOA). B) Diagram of the vectors used to target the second allele and LOA to confirm GYPC-/- clones.
(TIF)

S3 Fig Confirmation of inactivation of Glycophorin C. A) Glycophorin C transcript accumulation by qRT-PCR, in wild type (WT) cells upon differentiation (Diff) compared to the GYPC-/- clone. B) Western blot of differentiated JM8 cells WT and two knock out clones for GYPC 37 and 89.
(TIF)

S4 Fig Genotyping of the Slc4a1 edited clones. Sequence shows defects in red and traces confirm the damage caused.
(TIF)

S5 Fig Confirmation of inactivation of Slc4a1 (Band-3). A) Band 3 transcript accumulation in wild type (E14) and Slc4a1-/- (SLC) cell lines at the pluripotent (E14, SLC), embryoid body (EB) and differentiated (Diff) stages using 2 sets of primers, one located upstream the critical region (SLC3) and one downstream (SLC5). B) Western blot od differentiated E14 cells WT and two knock out clones D06 and F06.
(TIF)

S6 Fig Invasion assay with labelled Slc4a1 differentiated cells and mCherry-expressing *P*. *berghei*. Differentiated E14 cells, wild type and two knock out clones for Band-3 were labelled with the cell tracker DFFDA-SE prior to infection with mCherry-expressing *P*. *berghei* parasites. The time points of 6 and 24 hours were followed and analysed by flow cytometry.
(TIF)
