# Supplementary figures and images for: A Stem Cell Strategy Identifies Glycophorin C as a Major Erythrocyte Receptor for the Rodent Malaria Parasite Plasmodium berghei
Source: PLoS One. 2016 Jun 30;11(6):e0158238. doi: 10.1371/journal.pone.0158238 (PMC4928779; doi:10.1371/journal.pone.0158238)

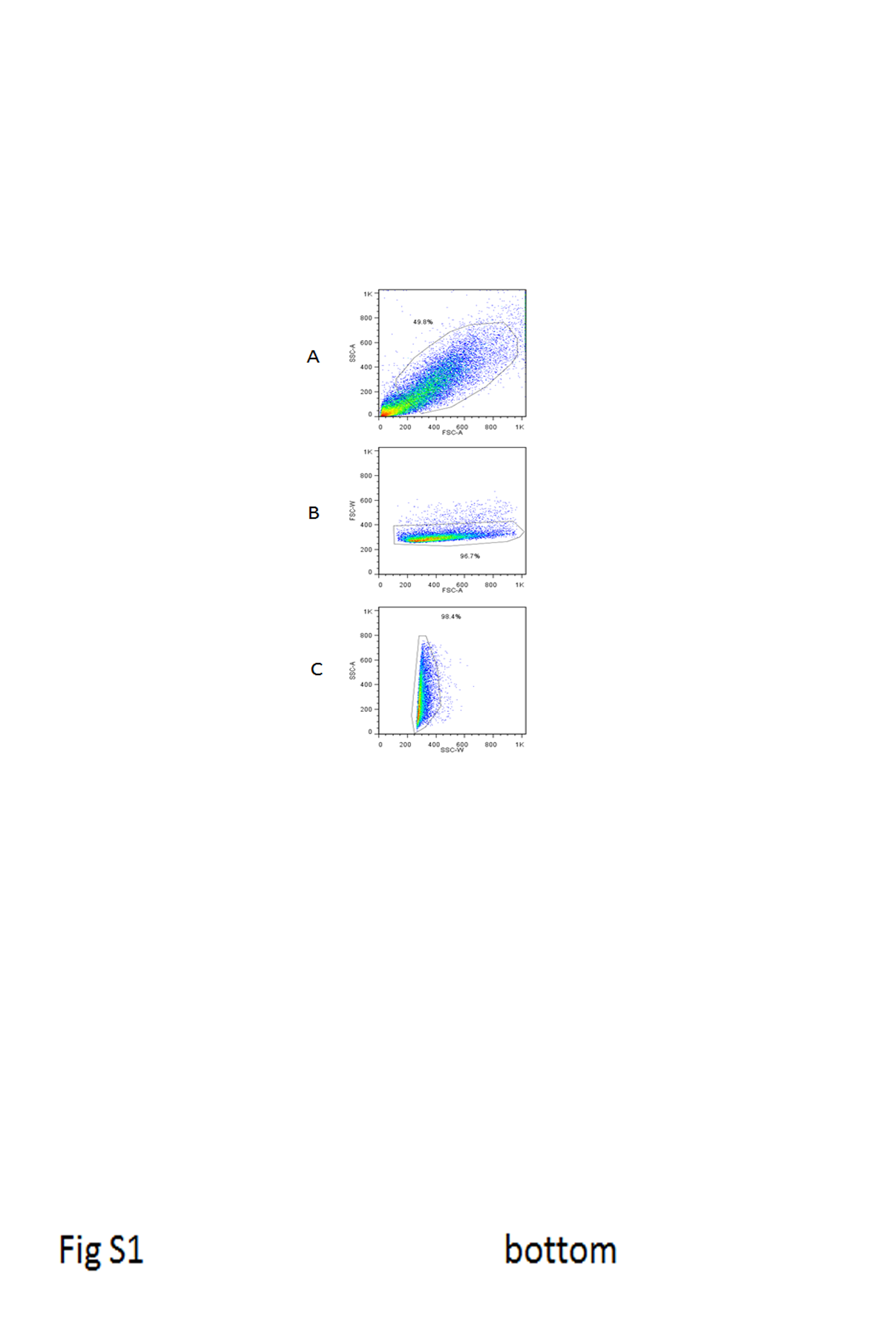

Supplement: S1 Fig — A) Debris was eliminated using SSC-A/FSC-A scatter plots. Doublets were excluded using B) FSC-W/FSC-A and C) SSC-A/SSC-W plots. (TIF) [file pone.0158238.s001.tif]

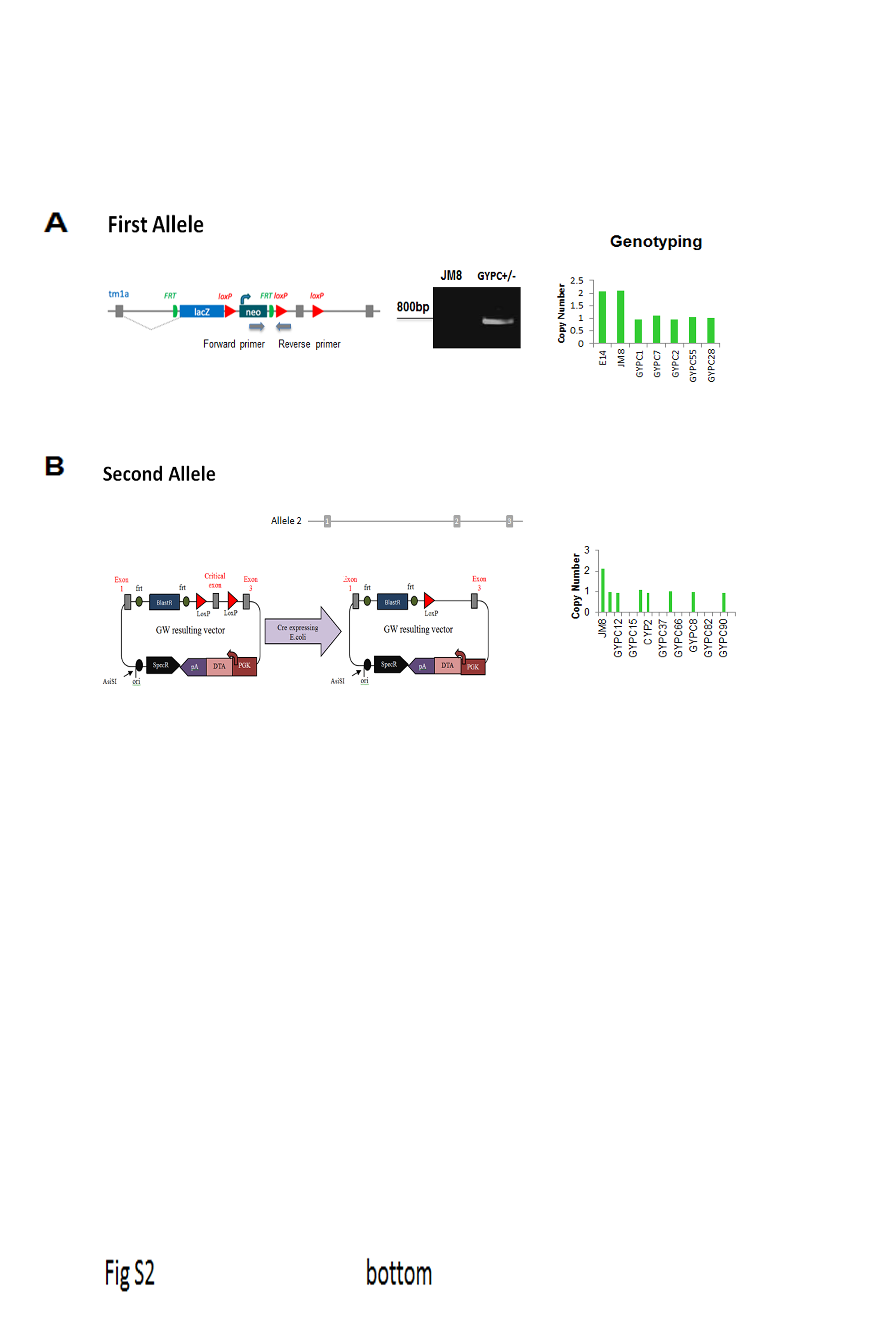

Supplement: S2 Fig — A) Diagram of the vector used to target the first allele. The colonies recovered were tested for vector integration by PCR, using the indicated primers. The copy number of the gene of interest was determined by Loss Of Allele assay (LOA). B) Diagram of the vectors used to target the second allele and LOA to confirm GYPC-/- clones. (TIF) [file pone.0158238.s002.tif]

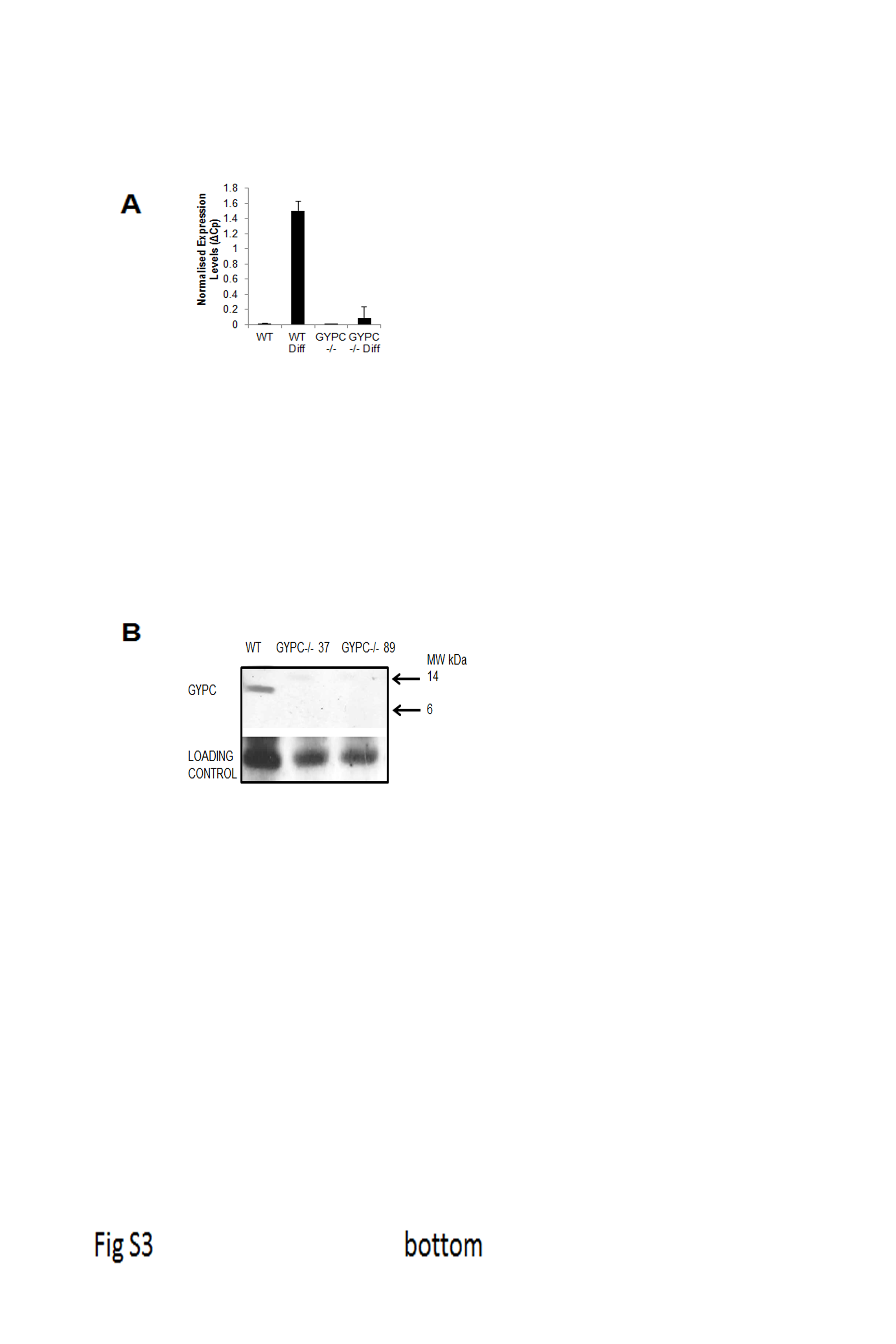

Supplement: S3 Fig — A) Glycophorin C transcript accumulation by qRT-PCR, in wild type (WT) cells upon differentiation (Diff) compared to the GYPC-/- clone. B) Western blot of differentiated JM8 cells WT and two knock out clones for GYPC 37 and 89. (TIF) [file pone.0158238.s003.tif]

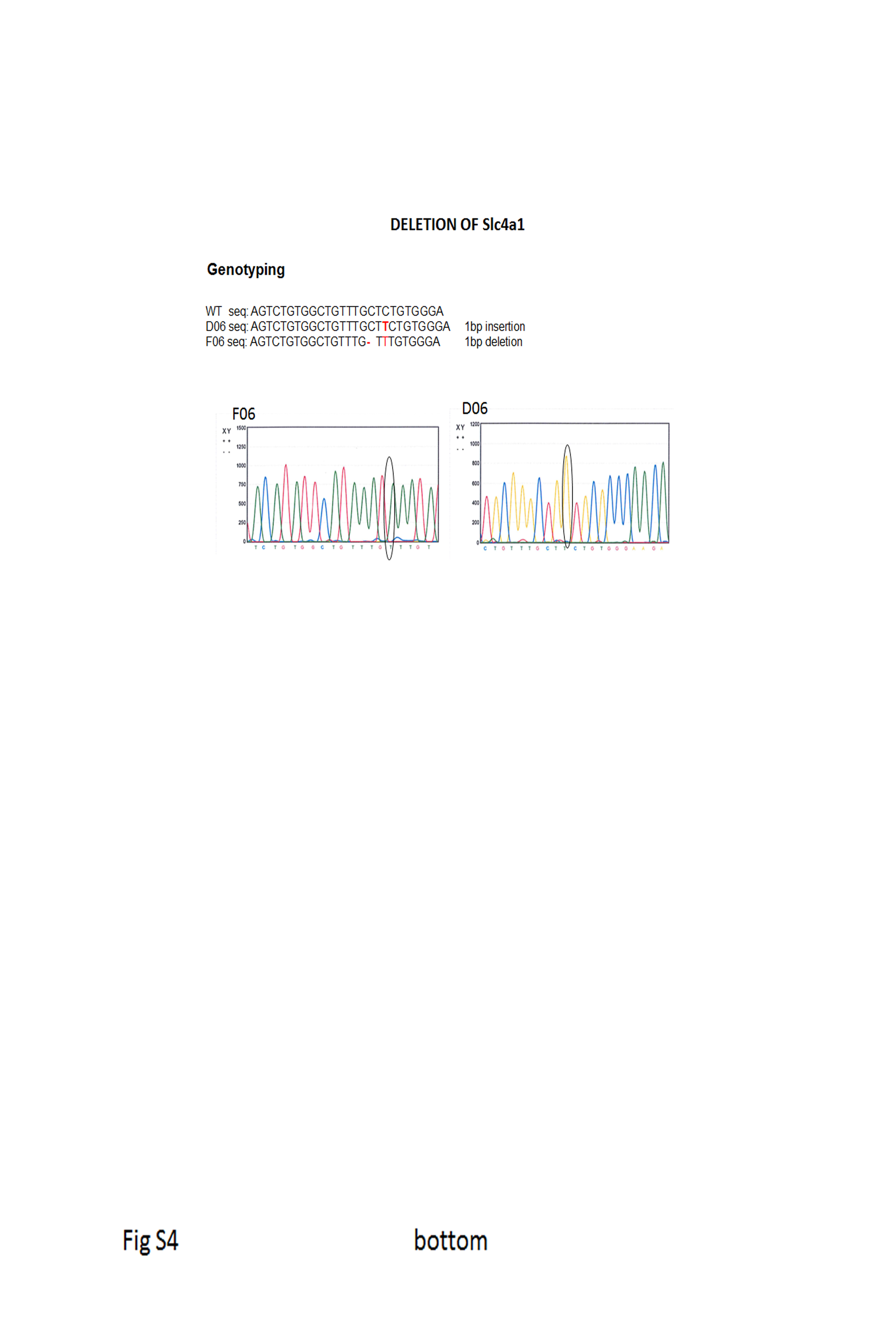

Supplement: S4 Fig — Sequence shows defects in red and traces confirm the damage caused. (TIF) [file pone.0158238.s004.tif]

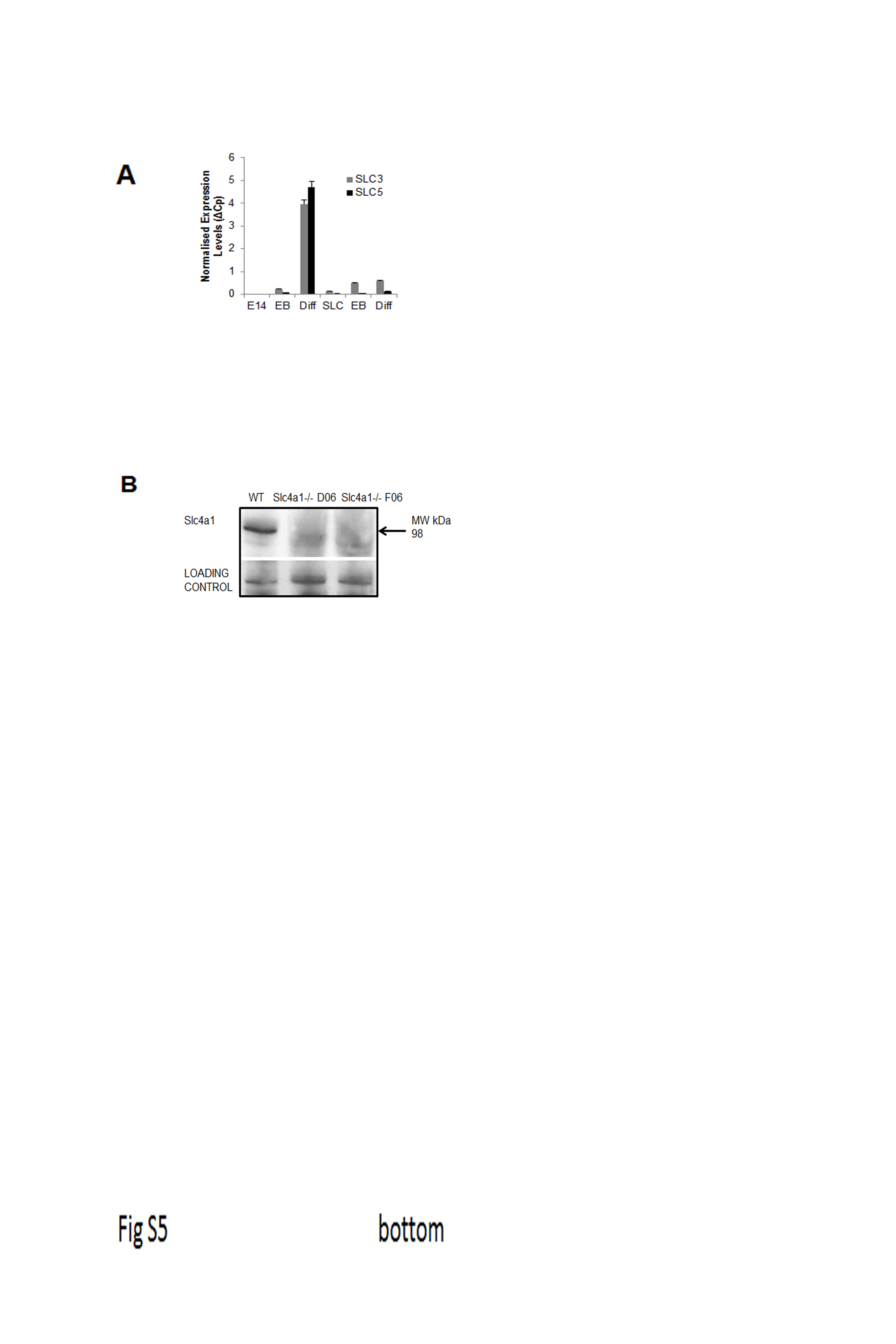

Supplement: S5 Fig — A) Band 3 transcript accumulation in wild type (E14) and Slc4a1-/- (SLC) cell lines at the pluripotent (E14, SLC), embryoid body (EB) and differentiated (Diff) stages using 2 sets of primers, one located upstream the critical region (SLC3) and one downstream (SLC5). B) Western blot od differentiated E14 cells WT and two knock out clones D06 and F06. (TIF) [file pone.0158238.s005.tif]

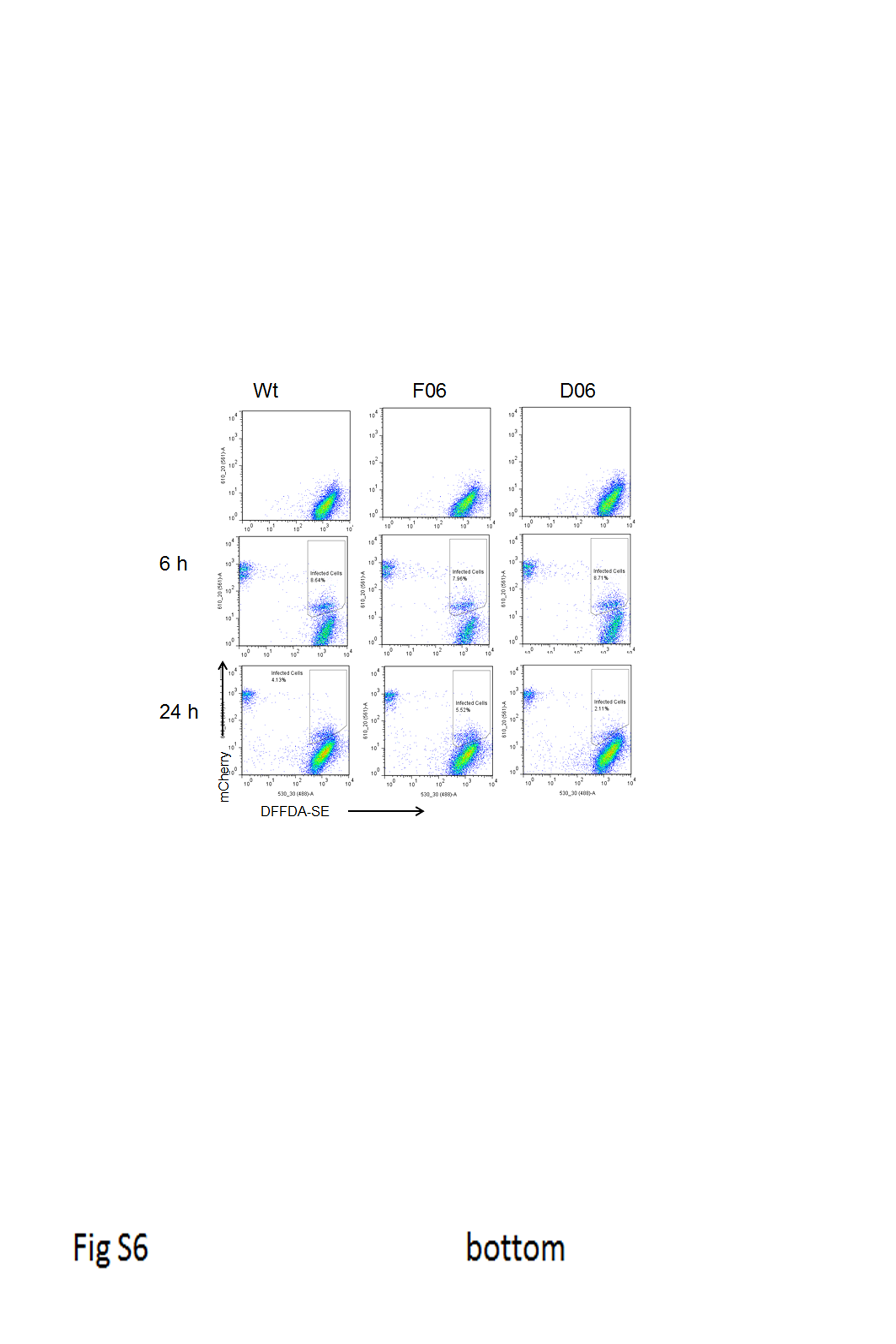

Supplement: S6 Fig — Differentiated E14 cells, wild type and two knock out clones for Band-3 were labelled with the cell tracker DFFDA-SE prior to infection with mCherry-expressing P. berghei parasites. The time points of 6 and 24 hours were followed and analysed by flow cytometry. (TIF) [file pone.0158238.s006.tif]
